# Trace Element Analysis in Whole Blood and Plasma for Reference Levels in a Selected Queensland Population, Australia

**DOI:** 10.3390/ijerph18052652

**Published:** 2021-03-06

**Authors:** Tatiana Komarova, Daniel McKeating, Anthony V. Perkins, Ujang Tinggi

**Affiliations:** 1 Queensland Health Forensic and Scientific Services, Coopers Plains, QLD 4108, Australia; tatiana.komarova@health.qld.gov.au; 2 School of Medical Sciences, Griffith University, Parklands Drive, Southport, Gold Coast, QLD 4222, Australia; d.mckeating@griffith.edu.au (D.M.); a.perkins@griffith.edu.au (A.V.P.)

**Keywords:** trace elements, reference range values, whole blood, plasma, serum, age group

## Abstract

The levels of trace elements in whole blood and plasma have been widely used for assessing nutritional status and monitoring exposure and can vary widely in populations from different geographical regions. In this study, whole blood samples (*n* = 120) and plasma samples (*n* = 120) were obtained from healthy donors attending the Red Cross Blood Bank (Queensland Red Cross Blood Service), which provided information for age and sex. There were 71 males (age range: 19–73 years) and 49 females (age range: 18–72 years) for whole blood samples, and 59 males (age range: 19–81 years) and 61 females (age range: 19–73 years) for plasma samples. The main aim of the study was to provide information on blood reference levels of 21 trace elements (Ag, Al, As, Bi, Br, Cd, Co, Cr, Cu, Hg, I, Mn, Mo, Ni, Pb, Sb, Se, Tl, U, V, Zn) in Queensland. The study also aimed to assess differences in trace element blood levels between males and females and the effect of age. The trace element levels in blood samples were analysed using inductively coupled plasma mass spectrometry (ICP-MS) and the standard reference materials of Seronorm (Trace Elements Whole Blood) and UTAK (Trace Elements Serum) were used for quality control and assurance. The study found wide variations of trace element levels in whole blood and plasma, and generally the levels were comparable to other countries. No detectable levels were found for Bi, Cr, U and V in whole blood, but V levels were found in plasma samples. There were significant differences between males and females for whole blood Cu (*p* < 0.001), I (*p* = 0.009), Tl (*p* = 0.016) and Zn (*p* = 0.016). Significant differences were also found for plasma Cu (*p* < 0.001) and Se (*p* = 0.003) between males and females. There were trends of increased levels of blood Pb, Se and Zn with age. The study has provided further information on a wide range of trace elements in blood as reference levels for Queensland and Australia which are currently lacking.

## 1. Introduction

Trace elements are naturally occurring in the Earth’s crust. However, a rapid increase in industrial and agricultural activities over the last few decades has contributed greatly to the distribution of trace elements into the environment. These trace elements will accumulate in the food chain and subsequently a major route of exposure to humans. Increased exposure to trace elements, particularly the toxic metals, has caused concern in human populations. The adverse health effects of metals such as mercury and lead which can cause neurotoxicity or kidney disease from cadmium are well documented [1,2]. Some trace elements are essential for humans and deficiency diseases such as impaired thyroid metabolism from low iodine, anaemia from low iron, and cardiomyopathy from low selenium are well documented [3,4].

There has been increased concern about high exposure of trace elements in various populations and this has led to many countries regularly monitoring the levels in human blood, urine, hair and toenails [5,6,7,8,9,10]. Whole blood samples are frequently used to monitor the levels of trace elements for assessing health status. The levels in whole blood tend to indicate recent and long-term exposure of toxic metals such as lead and cadmium. The main route of exposure of metals in humans includes ingestion of food and water, dermal absorption and inhalation of dust emissions. The levels of trace elements can vary significantly in a population and between geographical regions, and this can depend on dietary habits, occupational environment, lifestyles and genetic variations [11,12].

To the best of our knowledge, there has been no recent study investigating a wide range of trace elements in the Queensland population from various regions and among age groups, sex and health status. However, there have been studies on levels of individual elements such as lead in children blood in Mt. Isa residents and selenium in selected Queensland populations of different ages [13,14,15]. Our work is the first study providing the reference range values of essential and non-essential trace elements in blood and plasma of a selected cohort of adults in Queensland, Australia. The study will fill a current knowledge gap and provide important information for health workers. The data could be used as a guideline for health assessment from toxic element exposure and evaluation of nutritional status from essential trace element deficiency.

The ICP-MS analytical technique is increasingly being used as a method of choice in many laboratories for analysis of a wide range of trace elements (20–50 elements) in various biological sample matrices including blood and plasma [16,17]. The ICP-MS is a powerful analytical tool that allows multi-element determination from the same sample with high analytical sensitivity. However, there are still challenges for analysis of ultra-low trace element levels (< ng/L) from complex sample matrices which can contain high levels of various elements and may cause elemental interactions and polyatomic interferences during analysis [18,19]. There is also an increased interest in developing methods for low sample volume of clinical samples such as blood for trace element analysis [20,21].

The aim of this study was to conduct analysis of trace element levels in blood samples of selected Queensland population using ICP-MS method to provide reference range values for a wide range of trace elements in adults and to determine the differences of trace element levels between age groups and genders.

## 2. Materials and Methods

### 2.1. Equipment and Apparatus

An inductively coupled plasma mass spectrometer Agilent ICP-MS 7700 (Agilent Technologies, Tokyo, Japan) was used for determination of trace elements in plasma and blood samples. The instrument was equipped with Agilent ASX-500 Series ICP-MS Autosampler (Agilent Technologies, Victoria, Australia) and Integrated Sample Introduction System (ISIS) (Agilent Technologies, Victoria, Australia) for sample introduction.

Dispenser (Hamilton Microlab Dispenser 600 series, Merck) was used to add ultrahigh purity deionised water during blanks, standards and Quality Controls preparation and samples dilution. All samples were homogenised on Ratek Roller Mixer (Ratek Instruments, Victoria, Australia) before taking a required volume for analysis. Blood samples were mixed with alkaline solution using Vortex Mixer (Ratek Instruments, Victoria, Australia).

To prevent contamination, all labware such as 10 mL plastic tubes (Sarstedt Australia, Mawson Lakes, Australia) and caps for blanks (Sarstedt Australia, Mawson Lakes, Australia), standards, samples and Quality Controls preparation were acid-washing by soaking in 10% Nitric acid for at least 24 h before use.

Calibrated pipettes (Eppendorf Multipette E3x, Socorex 100–1000 µL, Finnpipete 100–1000 µL) (Sarstedt Australia, Mawson Lakes, Australia) were used during blanks, standards, quality controls and samples preparation procedures.

### 2.2. Reagents

Reagents for the preparation of alkaline solution: Triton X100 (1%) (commercially available detergent, Sigma-Aldrich), 2-propanol/isopropanol (AR Grade, Sigma-Aldrich), ammonia solution (25% Suprapur, Merck), ethylenediaminetetraacetic acid (EDTA, Sigma-Aldrich), ultrahigh purity deionised water (DI water, <2 MΩ × cm at 25 °C) produced by water treatment system (Aqua Cure, Burscough, UK).

Reagents for the preparation of standards (high-purity standards from Choice Analytical): Multi-Element Stock Standard Solution containing 10 mg/L of Ag, Al, As, Bi, Cd, Co, Cr, Cu, Mn, Mo, Ni, Pb, Sb, Se, Tl, V and Zn and Single Element Stock Standard Solutions of Hg (10 mg/L), I (10 mg/L), Br (1000 mg/L), Cu (1000 mg/L) and Zn (1000 mg/L)

Reagents for the preparation of internal standard solution for on-line addition (high-purity standards from Choice Analytical): scandium (Sc) (1000 mg/L), rhodium (Rh) (1000 mg/L), tellurium (Te) (1000 mg/L) and iridium (Ir) (1000 mg/L). Extra whole blood and plasma samples supplied by the Blood Bank were used as blank blood and blank plasma solutions for calibration and matric correction.

### 2.3. Certified Reference Materials for Quality Control

The commercially available Certified Reference Materials were used as quality controls (QCs) in the ICP-MS analysis of blood and plasma samples after reconstituting as per manufacturer’s instructions: Seronorm Trace Elements Whole Blood L-1 (SRM1) and Seronorm Trace Elements Whole Blood L-2 (SRM2) (Elitech); UTAK Trace Elements Serum Control Normal Range (UTK1) and UTAK Trace Elements Serum Control High Range (UTK2) (PM Separations). Two sets of quality controls of SRM1 and SRM2 for blood samples and UTK1 and UTK2 for plasma samples were determined at the beginning of the run and end of each run of 20 samples.

### 2.4. Sample Collection

The whole blood samples (*n* = 120) collected in EDTA (purple) blood tubes and serum (plasma) samples (*n* = 120) were obtained from the Red Cross blood bank, Queensland, in agreement with the Griffith University (Material Supply Deed N: 18-04QLD-13). In addition, an institutional clearance to analyse human blood was approved by Griffith University (HREC 2016/423) and Mater Medical Research (Mater Misericordia HREC/MML/64703). Samples were donated by healthy subjects who passed the health screening process prior to sample collection at Red Cross. The samples were collected between the period of 4 months from July to November 2018. The samples were then de-identified and the only information provided by the Red Cross blood bank was age and gender of the donors. The summary statistics of blood and plasma samples from the Red Cross blood bank are shown in Table 1.

### 2.5. Sample Preparation and Analysis

All samples were placed on Ratek roller mixer to ensure complete homogenisation of samples. An aliquot of 0.25 mL of each sample was taken and added into a 10 mL acid washed tube with 0.5 mL alkaline solution and made up to 5 mL final volume (20-fold dilution). The sample solution was placed on Vortex Mixer for thorough mixing and decomposition. Alkaline solution was prepared in a 500 mL plastic bottle by adding EDTA (5 g), 50 mL ammonia solution, 50 mL isopropanol and 5 mL Triton X-100

The analyses of trace element levels in whole blood and plasma samples were conducted at the Inorganic Chemistry laboratory of Queensland Health Forensic and Scientific Services (QHFSS), a NATA (National Association of Testing Authorities) accredited laboratory. The operating conditions of ICP-MS are shown in Table 2.

### 2.6. ICP-MS Analysis and Conditioning Run

#### 2.6.1. Calibration Standard Solutions

Calibration standard solutions were prepared by transferring the corresponding volumes of intermediate standard solutions into 10 mL tubes. The intermediate standard solutions were prepared from the Multi-Element and Single Element Stock Standard Solutions after making serial dilutions with DI water. All calibration standards containing 1 mL alkaline solution was made up to 10 mL final volume. All blood calibration standards were prepared to contain 0.5 mL Blank Blood. However, for plasma calibration standards, no blank plasma was added. The concentrations of calibration standard solutions ranged from 0.1 to 100 µg/L for Ag, Al, As, Bi, Cd, Co, Cr, Mn, Mo, Ni, Pb, Sb, Se, Tl and V, from 0.1 to 1000 µg/L for Cu and Zn, from 0.1 to 1 µg/L for Hg and from 5 to 1000 µg/L for I and 50 to 10,000 µg/L for Br.

#### 2.6.2. Blanks and Calibration Blanks

At least seven blank solutions were used in each run. They were prepared by adding 1 mL of alkaline solution to an acid washed 10 mL tube with 9 mL DI water. Blood calibration blank was prepared to contain 1 mL alkaline solution, 0.5 mL blood blank and 8.5 mL DI water. Plasma calibration blank did not contain plasma and had 1 mL alkaline solution and 9.5 mL DI water.

#### 2.6.3. Clinwashes Solutions

The Clinwashes solutions were prepared in a 50 mL digestion vessel by adding 5 mL alkaline solution, 2.5 mL either blank blood or blank plasma and 42.5 mL DI water. Plasma and blood Clinwashes solutions were used to condition the ICP-MS sample delivery system and sample cone before calibration (blood analyses) or after calibration (plasma analyses) and used as washes to clean the system during ICP-MS runs.

#### 2.6.4. Internal Standard Solution

Internal standard solution (for on-line addition) was prepared to contain 0.5% nitric acid, 0.5% hydrochloric acid and high purity standards of Sc, Rh, Te and Ir at concentrations 5, 0.4, 4 and 0.4 mg/L, respectively.

#### 2.6.5. Preparation of Quality Controls and Spikes

Certified Reference Materials (CRMs) were reconstituted as per manufacturer’s instructions. Then, 0.25 mL of each CRMs was added into a 10 mL tube with 0.5 mL alkaline solution and made up to 5 mL with DI water for a final 20-fold dilution factor.

A spiked sample included in each batch was prepared by spiking the sample with all elements prepared from the intermediate standard solutions with appropriate concentrations for each element. At least one replicate sample was run for every 20 samples.

### 2.7. Statistical Analysis

The data analysis was carried out using GraphPad Prism v 8.4.1 (GraphPad Software, San Diego, CA, USA) and R version 4.0.2 (R Foundation for Statistical Computing Platform, Vienna, Austria). Results are reported as mean (range), median and percentiles range from P2.5 to P97.5. For statistical purposes and analyses, the concentrations of trace elements with levels <LOR (mg/kg) were taken as LOD mg/kg. Others have used LOD/2 or LOQ/2 (limit of quantification/2) for data evaluation [8,18,21]. The levels of trace elements were non-normally distributed. Therefore, the non-parametric statistics of Mann–Whitney U Test was used for analysis of significant differences between groups (sex, age). Any difference between groups at *p* < 0.05 was considered to be statistically significant.

## 3. Results

### 3.1. Quality Control and Assurance

The levels of trace elements in Quality Controls were within the range of the reference values of the Seronorm Trace Elements Whole Blood (Table 3), except for Br and I which were consistently lower for all ICP-MS analysis of blood samples. Concentrations of Ag, Bi, Cr, Ni, and Tl in Seronorm Whole Blood L-1 were below the method detection limits. The levels of trace elements in the UTAK Trace Element Serum Controls were within the range of the reference values. The expected concentrations of Bi, Tl and V were below the detection limits of the method.

The recoveries of trace elements from the spiked blood samples ranged from 96% to 107% and from 95% to 108% for plasma samples. Slightly high recovery of Se (121%) was found in spiked plasma samples (Table 4). However, the concentrations of Se in UTK1 and UTK2 were within the expected ranges (Table 3). Spike recovery for Hg in whole blood samples is characterised by wide variation (Table 4). This can be related to the use of a solution with quite low and unstable level of Hg (0.5 µg/L) as a spike. Concentrations of Hg found in SRM1 and SRM2 complied with the certified values (Table 3).

Our Inorganic Chemistry laboratory has also regularly participated in Quality Assurance Programs organised by The Royal College of Pathologists of Australasia (RCPAQAP), and our results were within the acceptable ranges for all requested trace metals.

### 3.2. Trace Element Levels in Blood

The levels of trace elements in whole blood and plasma varied widely and the results are summarised in Table 5. The levels of Bi, Cr, U and V in blood samples were below the limits of detection of the method. The values of trace element limits of detection (LOD) and limits of reporting (LOR) for the method are shown in Table 5. The LODs were calculated from a series of blank samples (*n* = 21) analysed in different days as three times the standard deviation of the blanks multiplied by sample dilution factor of 20. LORs were calculated as three times LODs.

The trace element levels between females and males in whole blood and plasma samples were evaluated using non-parametric Mann–Whitney U Test for statistical differences. There were significant differences for levels of Cu (*p* < 0.001), I (*p* = 0.009), Tl (*p* = 0.016) and Zn (*p* = 0.016) in whole blood with higher levels of Cu and I, but lower levels of Tl and Zn in females in comparison to males (Table 6). There were significant differences between sexes for Cu (*p* < 0.001) and Se (*p* = 0.003) in plasma, with higher Cu level (1240 μg/L) and lower Se level (124 μg/L) in females compared to males (Table 6).

The median age of the Australian population is approximately 40 years old, and this median age is used to divide the participants into groups of under 40 years and over 40 years of age [22]. The results showed significant differences between groups for whole blood levels of Ag (*p* = 0.034), Hg (*p* = 0.031), Pb (*p* = 0.003), Se (*p* = 0.026) and Tl (*p* = <0.001) (Table 7). A generalised linear regression was used to evaluate correlations and trends between blood trace element levels and the age of the participants (Figure S1).

There were some correlations between age and blood Hg, Pb, Se and Zn which indicated a trend of increased levels of these elements in whole blood with age (Figure S1). Significant correlations were also observed between age and plasma Se and Cu with increased trend for Se with age and decreased trend for Cu.

The results of whole blood and plasma concentrations were presented as geometric means (GM) for the percentile ranges (Table 8). This would provide meaningful ranges when the impact of any outliers was reduced for percentile ranges. The results in Table 8 also show that trace element blood levels between males and females are generally comparable, except for Cu and Br which were relatively higher in females than males.

## 4. Discussion

The availability of data on baseline levels of a wide range of trace elements in whole blood and blood plasma/serum as reference values in a population is important information for nutritional and clinical monitoring of potential exposures. Due to increased exposure to trace elements including toxic elements, there have been a number of studies to establish blood trace element reference ranges in France, Canada, Korea and Germany [8,9,18,23]. These investigations have shown a wide variation of trace elements in human blood. The data of trace element levels in whole blood and blood plasma from various countries are shown in Tables S1 and S2. Therefore, there have been no studies on the analysis of a wide range of trace elements in blood of healthy population in Queensland, Australia.

### 4.1. Non-Essential Trace Elements

Exposure to non-essential trace elements, which have no biological functions in human body and can be toxic such as Pb, Hg and Cd, is a health concern in a workplace. Wide variations for non-essential trace elements such as Ag (<0.1–0.82 μg/L), As (<0.2–41.6 μg/L), Br (2820–12,200 μg/L), Hg (<0.8–9.3 μg/L) and Pb (3.8–49.6 μg/L) were observed in whole blood in the present study (Table 5). Relatively low levels of Sb and Tl were found in blood of Queenslanders (Table 5). Low levels of blood Sb and Tl have been reported for a population in Sweden and Germany [18,24]. As the blood levels of Bi, Cr, U and V were below the limit of detection of the method the reference values for these elements were not established. The levels of V in plasma were low (0.2–0.22 μg/L), and low plasma V levels (0.06 ± 0.03 μg/L) have also been reported for a healthy urban Italian population [25]. The Ag blood levels (<0.1–0.82 μg/L) were also low and comparable to Ag levels (<0.017–0.4 μg/L) of German adults [18]. As for plasma Al levels, two values (22 and 82 μg/L) were unexpectedly high and when measured at percentile ranges by gender (Table 8), the levels were within percentile ranges reported for Swiss and Italian adults [25,26].

The high level of As (42 μg/L) was found in one of the females in this study (Table 6). The high As blood levels (0.9–59.8 μg/L) have been shown for a population of Korean women [27]. The elevated levels of As in blood could be potentially attributed to a high intake of seafood and seaweed which can contain As as harmless compounds of arsenobetaine and arsenosugars [11,28]. Several studies have reported the association of elevated levels of As and Hg in blood and urine with high intake of seafood and fish [29,30,31,32]. In this study the blood Hg levels for men (<0.8–7.7 μg/L) and women (<0.8–9.3 μg/L) were relatively low and comparable to Hg levels for women (0.33–11.0 μg/L) and men (0.32–14.5 μg/L) in a Finnish population [33]. The blood Hg levels were observed to increase with age, and the over 40 years age group was significantly higher (*p* = 0.031) in mercury than the under 40 years age group (Table 7, Figure S1).

There has been little information on the levels of Br in human blood, and surveys of trace element levels in many countries have not reported reference values for Br. In this study the blood Br levels varied widely for men and women, and the overall mean (4960 μg/L) and range (2820–12,180 μg/L) of all sexes were comparable to the blood Br levels (mean: 5300 μg/L, range: 2500–11,700 μg/L) reported in earlier studies in Queensland, Australia [34]. Relatively low Br levels in blood (3200–5600 μg/L) have been reported for 10 healthy adults of North Carolina residents and Chinese adults (1050–3600 μg/L) [35,36]. Analysis of Br in blood is often associated with concern of occupational exposure from brominated hydrocarbons such as halothane and methyl bromide in the workplace, and elevated blood Br levels greater than 66,000 μg/L have been observed for workers in some environments [37].

As a toxic heavy metal, Pb exposure is a major concern in the workplace, and its blood levels in a population of high-risk groups are regularly monitored in many countries. Pb is known to have strong affinity for erythrocytes, therefore its levels in the whole blood are widely used for assessment of health effects and clinical intervention [38,39]. In this study, the blood Pb levels showed wide variations for males (4.9–45.0 μg/L) and females (3.8–49.6 μg/L) (Table 6) and were comparable to the reference range values reported for healthy adults in USA and France [8,27] (Table S1). Relatively low levels (<5–25.5 μg/L) of blood Pb have been reported for pregnant women from Western Australia [40], and the levels are consistent with values obtained in this study. The overall mean and reference range values of blood Pb levels (13.6 μg/L; 3.8–49.6 μg/L) in this study were lower than 50 μg/L (5 μg/dL), the recommended threshold for notification and investigation in Queensland population [41]. Air pollution and emission from mining activities and smelters have been shown to contribute to elevated levels of blood Pb (19–224 μg/L or 1.9–22.4 μg/dL) in Queensland children [13,42]. The blood Pb levels tend to increase and accumulate with age, and the present study has shown that the age group over 40 years had blood Pb levels significantly higher (*p* = 0.003) than the age group under 40 years (Table 7, Figure S1). Other studies have also shown that blood Pb concentrations increase with age and tend to be higher in men than women which could be attributed to diet, working and living environments and lifestyle activities [43,44].

### 4.2. Essential Trace Elements

Numerous essential trace element in blood surveys have been carried out in many countries to assess nutritional status which often reflects the dietary patterns and health of the population. These surveys provide important information in assessing deficiency and excess of trace element status in the population and whether appropriate health intervention programs are required. The diseases associated with trace element deficiency such as cardiomyopathy from Se deficiency and hypothyroidism from iodine deficiency have been reported in some countries where appropriate intervention and elimination programs were undertaken [45,46,47]. The physiological mechanisms and the functional roles of essential trace element metabolism in human health outcomes have been widely reviewed [1,48,49]. In the present study, the blood levels of essential trace elements of Co, Cu, I, Mn, Mo, Se and Zn were found to vary widely. The levels of Co in whole blood (<0.2–1.1 μg/L) were comparable to German adults (0.04–0.8 μg/L) and its levels in plasma (0.21–1.3 μg/L) were relatively higher than that in the Swiss population (<0.1–0.155 μg/L) [18,26]. Whole blood or plasma (serum) is widely used for analysis of Cu and Zn because their levels in these matrices are generally high and can provide immediate information on health and nutritional status of individuals [50,51]. The blood Cu levels for females (710–1420 μg/L) and males (650–950 μg/L) in this study were comparable with Serbian and Korean populations [27,52]. The mean values of plasma Cu (1240 μg/L) and Zn (1140 μg/L) in females were consistent with mean values of Cu (1016 ± 375 μg/L) and Zn (896 ± 163 μg/L) reported for other Australian women [53].

As an essential trace element, Se plays important functional roles in antioxidant enzymes of glutathione peroxidases and thioredoxin reductases, and its levels in whole blood and plasma are widely used to assess health and nutritional status of people [54,55,56]. In this study, the blood Se levels (118–224 μg/L) were comparable to Se levels reported for adults in Brazil (68–245 μg/L) and Germany (85–182 μg/L) [18,57]. The overall mean of Se in whole blood (141 μg/L, Table 5) and plasma (130 μg/L, Table 5) were higher than the mean values (whole blood: 122 μg/L; plasma: 102 μg/L) reported in 2002 survey of the South Australian population [58]. This survey has also shown the decreasing trends from 1977 to 2002 as a result of decreased Se levels in wheat crops, a source of staple food [58]. The mean plasma Se value (130 μg/L) in this study was relatively higher than the mean value (110 μg/L) found in the southern Tasmanian population [59]. The overall plasma Se levels (mean: 130 μg/L, range: 82–180 μg/L) in our study were consistent with Se levels (mean: 85.6 μg/L, range: 55.2–173.7 μg/L) reported in Queensland population from three different regions earlier [15]. Another Queensland study indicated a decline in Se plasma levels with age, which was different from our data showing an increased trend of Se plasma levels with age [14] (Figure S1). The difference in findings of Se status with age could be explained by dietary patterns or/and intakes of vitamin and mineral supplements which have been shown to increase plasma Se levels [15,59]. In the present study, no information was available on dietary patterns and lifestyles of the participants.

There have been few data on the analysis of blood iodine levels in a population [36]. The use of ICP-MS was found to be effective and satisfactory for the blood I analysis in alkali condition. The levels of whole blood I vary widely among the age and gender groups in the Chinese populations ranging from 13.9 to 33.6 μg/L for adolescents and 14.1 to 812 μg/L for adults [36]. In this study, the overall mean (30.1 μg/L) and the range values (19.5–82.7 μg/L) of whole blood I were within the Chinese population range. Another study of the Chinese adults [60] reported the serum I interquartile range levels of 67.9–84.8 μg/L which were closer to the percentile ranges for females (P90–P97.5: 40.6–60.2 μg/L) and males (P90–P97.5: 34.7–37.8 μg/L) in this study (Table 8).

## 5. Conclusions

The findings of the present study have demonstrated wide variations of trace element levels in whole blood and plasma of Queensland population. Concentrations of some trace elements were below the LOD of the method. Low levels of Bi, Cr, U and V were found in blood samples. Generally, the trace element blood levels reported in this study were comparable with values found in other countries. The study has revealed significant differences of Cu, I, Tl and Zn in whole blood and Cu and Se in plasma between men and women and the trend of increased blood Pb, Se and Zn with age.

There have been few reports on the analysis of trace elements in blood of adult population of Queensland, Australia. The findings in this study have provided information on a wide range of trace elements in blood and plasma which could be used as reference ranges for assessing nutritional and health status of the Queensland population. Unlike the monitoring survey programs in USA with the National Health and Nutrition Examination Survey (HNANES) and Europe with EURO-Trace Element Reference Values in Human Tissue (TERVITH), there is no similar program in Australia to monitor wide range of trace elements in blood of a population for all ages from different geographical regions. A recently published preliminary study of two areas in Queensland with no Pb mines or smelters found low levels of blood Pb (4.1–41 µg/L) in age groups from 0.5 year to 37 years including pregnant women [61]. Excluding for blood Pb levels, there is little study to include a wide range of other trace element levels in the blood of Queensland population and across Australia, and this should be considered for future study to provide further information on baseline levels of blood trace elements.

## Figures and Tables

**Table 1:** Age and sex of blood bank donors.

Whole Blood Donor Age (Year)					Plasma Donor Age (Year)				
Sex	Mean	Median	Min	Max	Sex	Mean	Median	Min	Max
M (*n* = 71)					M (*n* = 59)				
F (*n* = 49)					F (*n* = 61)				
M and F	46.3	48	18	73	M and F	44.6	46	19	81
M	47.3	49	19	73	M	48.4	49	19	81
F	44.9	45	18	72	F	40.9	43	19	73

**Table 2:** ICP-MS operating conditions.

RF Power	1350 W
RF Matching	1.95 V
Sample Depth	8 mm
Nebulizer Gas	0.8 L/min
Nebulizer Pump	0.1 rps
Dilution Gas	0.25 L/min
Spray Chamber Temp.	2 °C
Peak Pattern	1 point
Replicates	3
He flow	4.5 mL/min
Sweeps/Replicate	100

**Table 3:** Mean levels of trace elements in the Certified Reference Materials of quality control for whole blood and plasma.

Element	Unit	Seronorm Whole Blood QC				UTAK Trace Element Serum Control			
		This Study	Whole Blood L-1	This Study	Whole Blood L-2	This Study	Normal Range	This Study	High Range
Ag	µg/L	<0.1	0.075	<0.1	0.073	-	-	-	-
Al	µg/L	-	11.6 (5.8–17.5)	-	68.9 (55.0–82.7)	13.0	10.9 (6.5–15.3)	192	201 (161–241)
As	µg/L	1.93	2.4 (1.9–29)	11.6	14.1 (11.3–17.0)	-	-	-	-
Bi	µg/L	<0.1	-	5.27	5.00 (3.99–6.00)	<0.08	-	<0.08	-
Br	µg/L	714	1053	693	980	-	-	-	-
Cd	µg/L	<0.8	0.28 (0.17–0.40)	4.86	5.01 (4.00–6.02)	-	0.17 (0.13–0.21)	-	9.0 (7.2–10.8)
Co	µg/L	0.21	0.20 (0.12–0.28)	5.01	5.18 (4.13–6.22)	0.27	-	0.29	-
Cr	µg/L	<5	0.45 (0.27–0.63)	9.4	10.7 (8.5–12.8)	0.49	0.16 (0.12–0.20)	2.42	2.27 (1.82–2.72)
Cu	mg/L	0.58	0.64 (0.51–0.76)	1.17	1.34 (1.07–1.60)	0.99	1.08 (0.81–1.35)	2.76	3.06 (2.4–3.7)
Hg	µg/L	1.45	1.48 (1.18–1.77)	17.1	17.0 (13.6–20.4)	-	-	-	-
I	µg/L	17.1	28.6 (22.8–34.3)	74	107 (86–129)	-	-	-	-
Mn	µg/L	17.3	18.4 (14.7–22.1)	29.8	31.4 (25.1–37.7)	0.56	0.47 (0.35–0.59)	2.47	2.51 (2.01–3.01)
Mo	µg/L	0.58	0.51 (0.41–0.61)	4.69	5.31 (4.24–6.37)	0.92	-	0.88	-
Ni	µg/L	<2	1.38 (1.10–1.66)	15.1	15.9 (12.7–19.1)	-	-	-	-
Pb	µg/L	10.1	9.9 (7.9–11.9)	324	337 (269–405)	-	-	-	-
Sb	µg/L	1.43	1.33 (1.06–1.60)	25.5	25.9 (20.7–31.1)	-	-	-	-
Se	µg/L	50	60 (48–72)	139	161 (128–193)	110	101 (76–126)	309	281 (225–337)
Tl	ng/L	<0.06	7 (3–11)	10.9	10.2 (8.1–12.2)	<0.04	-	<0.04	-
U	µg/L	0.21	0.18	0.19	0.18	-	-	-	-
V	µg/L	0.63	0.97 (0.58–1.35)	4.02	4.98 (3.98–5.98)	<0.20	-	<0.20	-
Zn	mg/L	4.2	(3.4–5.2)	7.2	7.1 (5.7–8.5)	0.69	0.68 (0.51–0.85)	2.57	2.64 (2.11–3.17)

**Table 4:** Spike recoveries of elements from whole blood and plasma.

Elements	Spike Level (µg/L)	Whole Blood Spike Recovery (%)	Plasma Spike Recovery (%)
		Mean ± s.d. (*n* = 3)	
Al	5	-	101.0 ± 3.4
Ag	5	100.5 ± 2.8	-
As	5	104.0 ± 5.6	-
Bi	5	101.2 ± 2.1	94.9 ± 1.8
Br	500	98.9 ± 3.0	-
Cd	5	99.5 ± 2.5	-
Co	5	97.9 ± 2.2	104.3 ± 1.8
Cr	5	96.0 ± 1.4	96.3 ± 1.4
Cu	505	101.0 ± 5.0	98.8 ± 1.5
Hg	0.5	120 ± 47	-
I	50	99.4 ± 1.4	-
Mn	5	101.1 ± 6.0	105.4 ± 3.6
Mo	5	97.9 ± 1.8	108.4 ± 1.0
Ni	5	98.5 ± 3.1	-
Pb	5	107.2 ± 15.2	-
Sb	5	96.7 ± 1.3	-
Se	5	100.5 ± 5.0	121.1 ± 6.2
Tl	5	97.6 ± 2.0	95.9 ± 1.8
U	5	98.2 ± 2.9	-
V	5	99.9 ± 2.9	99.1 ± 0.9
Zn	505	104.3 ± 10.5	103.7 ± 1.4

**Table 5:** The levels of trace elements in whole blood and plasma including method limit of detection (LOD) and limit of reporting (LOR).

Element (µg/L)	Whole Blood (*n* =120)	Plasma (*n* = 120)	Whole Blood		Plasma	
	Mean (Range) ^a^	Mean (Range)	LOD	LOR	LOD	LOR
Ag	0.19 (<0.1–0.82)	-	0.04	0.1	-	-
Al	-	6.9 (4–82)	-	-	1.2	4.0
As	2.2 (<0.2–41.64)	-	0.06	0.2	-	-
Bi	<0.1	<0.08	0.04	0.1	0.02	0.08
Br	4960 (2820–12,200)	-	18	52	-	-
Cd	0.80(<0.8–0.99)	-	0.2	0.8	-	-
Co	0.33 (<0.2–1.1)	0.47 (0.21–1.3)	0.06	0.2	0.02	0.08
Cr	<5	<1.7	1.8	5.0	0.6	1.7
Cu	840 (650–1420)	1100 (670–2490)	0.6	1.6	0.4	1.0
Hg	2.0 (<0.8–9.3)	-	0.2	0.8	-	-
I	30.1 (19.5–82.7)	-	0.4	1.5	-	-
Mn	9.7 (4.54- 19.5)	1.0 (<1–3.1)	0.2	0.8	0.4	1.0
Mo	0.49 (<0.3–1.7)	0.91 (0.26- 3.0)	0.2	0.3	0.08	0.2
Ni	2.0 (<2–4.0)	-	0.8	2.0	-	-
Pb	13.6 (3.8–49.6)	-	0.2	0.3	-	-
Sb	4.1 (2.6–6.2)	-	0.2	0.3	-	-
Se	141 (118–224)	130 (82- 180)	0.6	2.0	0.6	1.6
Tl	0.06 (<0.06–0.16)	0.11 (0.04–0.20)	0.02	0.06	0.02	0.04
U	<0.1	-	0.04	0.1	-	-
V	<0.45	0.2 (0.2–0.22)	0.2	0.45	0.06	0.20
Zn	6750 (4620- 9250)	1150 (820–1660)	4	11.5	1.6	5.0

^a^ For statistical purposes, the trace element concentrations < LOR (mg/kg) were taken as LOD (mg/kg) to determine for mean values.

**Table 6:** Trace element levels (mean and range) in whole blood and plasma of males and females.

Element (µg/L)	Whole Blood (*n* = 120)		Mann–Whitney U Test	Plasma (*n* = 120)		Mann–Whitney U Test
	Female (*n* = 49)	Male (*n* = 71)	Significance	Female (*n* = 61)	Male (*n* = 59)	Significance
Ag	0.20 (0.1–0.67)	0.17 (0.1–0.82)		-	-	
Al	-	-		6.4 (<4–21.8)	7.4 (<4–81.8)	
As	2.7 (0.2–42)	1.8 (0.2–13.0)		-	-	
Bi	<0.1	<0.1		<0.08	<0.08	
Br	5140 (2820–12,180	4830 (3120–9050)		-	-	
Cd	0.81 (<0.8–0.99)	0.80 (<0.8–0.93)		-	-	
Co	0.37 (<0.2–1.1)	0.30 (<0.2–0.86)		0.50 (0.22–1.34)	0.44 (0.21–1.1)	
Cr	<5	<5		<1.7	<1.7	
Cu	930 (710–1420)	770 (650–950)	*p* < 0.001	1240 (706–2485)	960 (672–1403)	*p* < 0.001
Hg	1.8 <0.8–9.3)	2.1 (<0.8–7.7)		-	-	
I	32.5 (20.1–82.7)	28.5 (19.5–44.6)	*p* = 0.009	-	-	
Mn	10.0 (5.7–17.6)	9.6 (4.5–19.5)		1.1 (<1–3.1)	1.0 (<1–1.3)	
Mo	0.51 (<0.3–1.7)	0.47 (<0.3–1.2)		0.85 (0.27–2.67)	0.96 (0.26–3.0)	
Ni	2.0 (<2–4.0)	<2		-	-	
Pb	13.2 (3.8- 49.6)	13.9 (4.9–45.0)		-	-	
Sb	4.0 (2.8–5.9)	4.1 (2.6–6.2)		-	-	
Se	140 (122–203)	142 (118–224)		124 (82- 179)	130 (101–161)	*p* = 0.003
Tl	0.061 (<0.06–0.10)	0.07 (<0.06–0.16)	*p* = 0.016	0.11 (0.05–0.20)	0.10 (0.04–0.20)	
U	<0.1	<0.1		-	-	
V	<0.45	<0.45		0.20 (<0.2–0.22)	0.20 (<0.2–0.22)	
Zn	6540 (4940–8390)	6900 (4620–9250)	*p* = 0.016	1140 (850–1660)	1160 (819–1496)	

Significant differences were found between female and male for whole blood Cu, I, Tl and Zn, and for plasma Cu and Se.

**Table 7:** Trace element levels in blood for under and over 40 years old.

Element (µg/L)	Whole Blood		Mann–Whitney U Test	Plasma		Mann–Whitney U Test
	Under 40 y (*n* = 43)	Over 40 y (*n* = 77)	Significance	Under 40 y (*n* = 45)	Over 40 y (*n* = 75)	Significance
Ag	0.17 (<0.1–0.65)	0.20 (<0.1–0.82)	*p* = 0.034	-	-	
Al	-	-		6.2 (<4–9.6)	7.4 (<4–81.8)	
As	1.53 (<0.2–13.4)	2.51 (<0.2–41.6)		-	-	
Bi	<0.1	<0.1		<0.08	<0.08	
Br	4740 (2820–9050)	5080 (3110–12,180)		-	-	
Cd	0.80 (<0.8–0.85)	0.81 (<0.8–0.99)		-	-	
Co	0.31 (<0.2–0.86)	0.34 (<0.2–1.1)		0.47 (0.21–1.2)	0.47 (0.21–1.34)	
Cr	<5	<5		<1.7	<1.7	
Cu	837 (649–1390)	840 (660–1420)		1223 (706–2490)	1030 (672–1963)	
Hg	1.52 (<0.8–6.71)	2.2355 (<0.8–9.3)	*p* = 0.031	-	-	
I	30.0 (19.8–42.7)	30.3 (19.5–82.7)		-	-	
Mn	10.2 (5.3–19.5)	9.5 (4.5–19.3)		1.0 (<1–1.3)	1.1 (<1–3.1)	
Mo	0.50 (0.3–1.7)	0.48 (0.3–1.2)		0.94 (0.29–2.67)	0.89 (0.26–3.0)	
Ni	2.0 (<2–4.0)	<2		-	-	
Pb	10.9 (4.8–31.4)	15.1 (3.8–49.6)	*p* = 0.003	-	-	
Sb	4.0 (2.8–5.1)	4.1 (2.6–6.2)		-	-	
Se	137 (119–156)	144 (118–224)	*p* = 0.026	124 (96–161)	129 (82- 179)	*p* = 0.013
Tl	0.07 (<0.06–0.11)	0.062 (<0.06–0.16)	*p* < 0.001	0.11 (0.05–0.20)	0.10 (0.04–0.16)	
U	<0.1	<0.1		-	-	
V	<0.45	<0.45		0.20 (<0.20–0.20)	0.20 (<0.20–0.22)	
Zn	6590 (4620–8650)	6840 (4910–9250)		1100 (820–1340)	1180 (890–1660)	*p* = 0.011

Significant differences were found between age groups (under and over 40 years) for whole blood Ag, Hg, Pb, Se and Tl, and for plasma Se and Zn.

**Table 8:** Trace element levels in whole blood and plasma as percentile range for females and males.

Element (µg/L)	Whole Blood Percentile Range							
	Female (*n* = 49)				Male (*n* = 71)			
	2.5–10%	25–75%	90–97.5%	GM ^a^	2.5–10%	25–75%	90–97.5%	GM
Ag	<0.1	0.10–0.25	0.31–0.61	0.17	<0.1	<0.1–0.21	0.26–0.44	0.15
As	<0.2	0.45–1.7	5.0–13.4	1.0	<0.2–0.25	0.46–1.73	3.5–12.1	0.95
Bi	<0.1	<0.1	<0.1	<0.1	<0.1	<0.1	<0.1	<0.1
Br	3140–3950	4520–5590	6380–6700	4990	3270–3500	4030–5610	5980–7890	4710
Cd	<0.8	<0.8	<0.8–0.86	0.81	<0.8	<0.8	<0.8	<0.8
Co	<0.2	<0.2–0.46	0.63–0.86	0.33	<0.2	<0.2–0.34	0.48–0.57	0.28
Cr	<5	<5	<5	<5	<5	<5	<5	<5
Cu	720–750	810–1020	1160–1390	920	660–680	730–810	850–940	770
Hg	<0.8	<0.81.9	3.3–6.7	1.4	<0.8	<0.8–2.6	4.9–6.8	1.6
I	22.6–25.8	26.1–34.0	40.6–60.2	31	20.6–22.9	25.2–30.9	34.7–37.8	28.1
Mn	6.6–7.3	8.0–11.5	13.0–13.9	9.7	5.2–6.2	7.0–11.3	13.4–18.7	9.1
Mo	<0.3	0.35–0.61	0.75–1.1	0.47	<0.3	0.3–0.54	0.77–0.98	0.43
Ni	<2	<2	<2	<2	<2	<2	<2	<2
Pb	4.5–5.5	8.0–15.3	24.3–33.4	11.2	5.4–6.7	9.2–16.5	19.6–33.4	12.5
Sb	3.0–3.2	3.4–4.3	4.7–5.0	3.9	2.9–3.4	3.7–4.5	5.0–5.8	4.1
Se	122–124	130–148	157–171	140	120–123	131–152	158–167	140
Tl	<0.06	<0.06	0.062–0.072	0.061	<0.06	<0.06–0.062	0.08–0.10	0.065
U	<0.1	<0.1	<0.1	<0.1	<0.1	<0.1	<0.1	<0.1
V	<0.45	<0.45	<0.45	<0.45	<0.45	<0.45	<0.45	<0.45
Zn	5250–5650	5910–7070	7660–8350	6490	5120–5740	6270–7480	8140–8570	6840
**Element (µg/L)**	**Plasma Percentile Range**							
	**Female (*n* = 61)**				**Male (*n* = 59)**			
	**2.5–10%**	**25–75%**	**90–97.5%**	**GM ^a^**	**2.5–10%**	**25–75%**	**90–97.5%**	**GM**
Al	<4–4.4	5.4–6.8	8.2–10.1	6.2	<4	4.7–6.9	8.2–12.0	6.2
Bi	<0.080	<0.080	<0.080	<0.08	<0.080	<0.080	<0.080	<0.08
Co	0.23–0.26	0.32–0.56	0.87–1.2	0.45	0.22–0.24	0.29–0.48	0.74–1.06	0.39
Cr	<1.7	<1.7	<1.7	<1.7	<1.700	<1.700	<1.700	<1.7
Cu	790–860	1000–1300	1870–2130	1190	710–790	860–1060	1130–1230	950
Mn	<1	<1	<1–1.3	1.0	<1	<1	1.1–1.2	1.0
Mo	0.35–0.46	0.54–1.1	1.3–2.0	0.77	0.31–0.47	0.63–1.1	1.7–2.1	0.85
Se	98–110	115–130	140–160	123	109–115	122–140	145–160	130
Tl	0.06–0.07	0.08–0.13	0.15–0.18	0.1	0.06–0.07	0.09–0.12	0.14–0.17	0.1
V	<0.2	<0.2	<0.2	<0.2	<0.2	<0.2	<0.2	<0.2
Zn	880–955	1020–1220	1300–1450	1130	910–990	1050–1260	1350–1460	1150

^a^ GM—geometric mean.

## Data Availability

The data presented in this study are available on request from the corresponding author. The data are not publicly available due to privacy of internal governmental report.

## Supplementary Materials

The following are available online at https://www.mdpi.com/1660-4601/18/5/2652/s1, Figure S1: Trends and correlations between trace element levels in blood, plasma and age, Table S1: Concentration of trace elements in human whole blood for both genders by country [8,18,27,33,44,52,57,62,63,64,65,66], Table S2: Concentration of trace elements in human blood plasma for both genders by country [24,26,27,52,67].
