# Supplementary material for: Trace Element Analysis in Whole Blood and Plasma for Reference Levels in a Selected Queensland Population, Australia
Source: Int J Environ Res Public Health. 2021 Mar 6;18(5):2652. doi: 10.3390/ijerph18052652 (PMC7967308; doi:10.3390/ijerph18052652)
Supplement: Supplementary file 1 [file ijerph-18-02652-s001.pdf]

International Journal of
Environmental Research and Public Health

MDPI

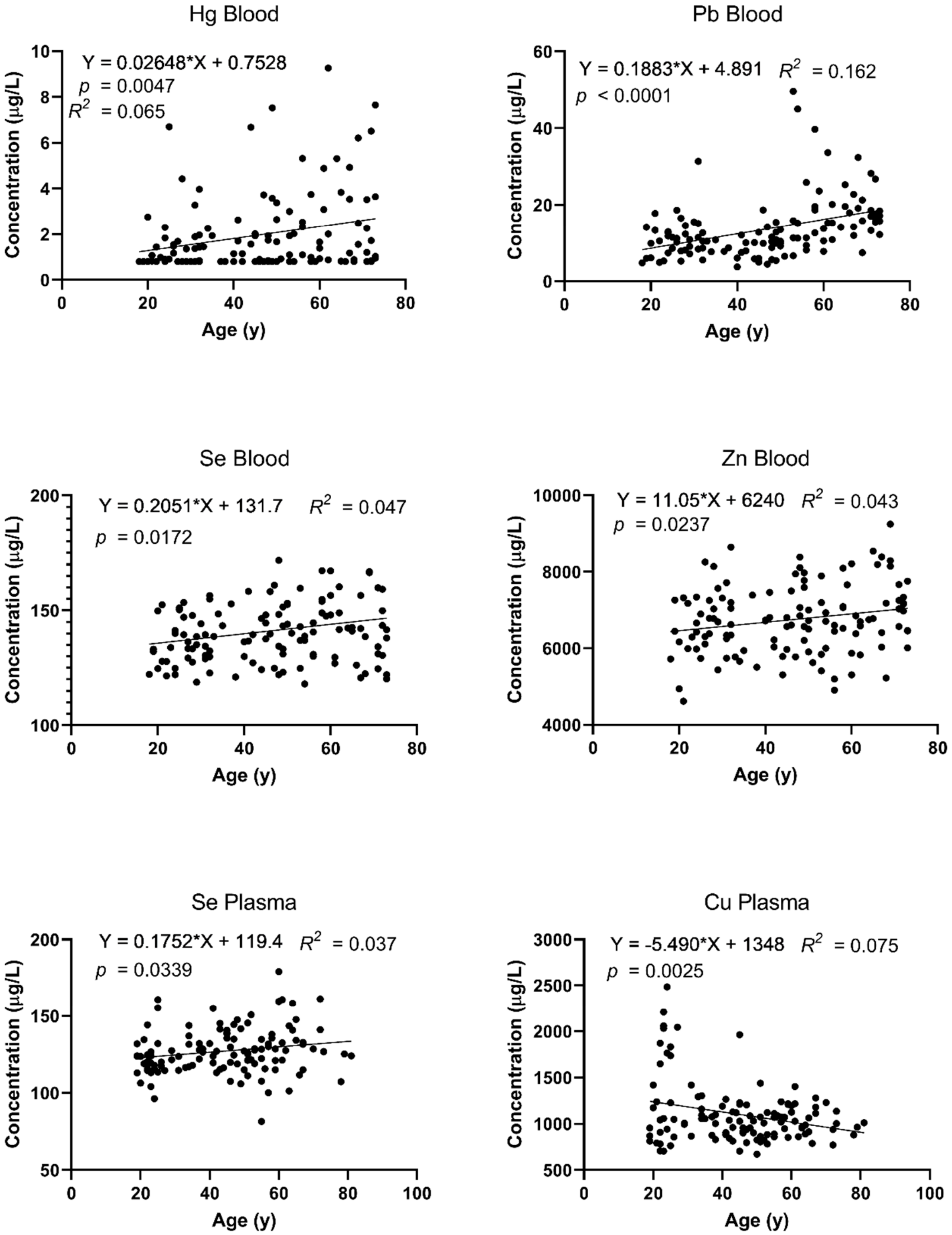

Supplementary Figure S1. Trends and correlations between trace element levels in blood, plasma and age.

Supplementary Table S1. Concentration of trace elements in human whole blood for both genders by country.

Element (μg/L)	Mean (range), female	Mean (range), male	Mean (range), (all sexes)	All age group (y)	Country	Reference
**Ag**	0.20 (0.1–0.67)	0.17 (0.1–0.82)	0.19 (<0.1–0.82)	18–73	Australia	This study
			0.072 (<0.017–0.4)	18–70	Germany	[18]
**As**	2.7 (0.2–42)	1.8 (0.2–13.0)	2.2 (<0.2–42)	18–73	Australia	This study
	0.96 (0.20–28.9)	0.83 (<LOD–22.4)		18–49	Slovenia	[62]
			1.1 (0.1–3.2)	18–60	Brazil	[57]
	10.02 (0.9–59.8)	10.85 (0.6–53.1)		12–78	Korea	[27]
			0.93 (0.13–4.2)	18–70	Germany	[18]
	2.69 (2.50–2.87)	2.55 (2.36–2.73)		20–59	France	[8]
	0.73 (0.08–5.92)	0.92 (0.08–18.02)		31.1 ± 0.34	Finland	[33]
**Bi**	<0.1	<0.1		18–73	Australia	This study
			<0.008 (<0.008–0.02)	18–70	Germany	[18]
**Br**	5140 (2820–12,180)	4830 (3120–9050)	4960 (2820–12,200)	18–73	Australia	This study
**Cd**	0.81 (<0.8–0.99)	0.80 (<0.8–0.93)	0.80(<0.8–0.99)	18–73	Australia	This study
	0.69 (0.46–0.99) [a]	0.68 (0.31–1.47) [a]		12–60	China	[63]
	1.00 (0.1–3.1)	0.83 (0.2–3.2)		12–78	Korea	[27]
	0.35 (<LOD-3.08)	0.23 (<LOD–4.80)		18–49	Slovenia	[62]
	0.4 (0.09–1.10)			18–60	Brazil	[57]
			0.57 (0.10–4.1)	18–70	Germany	[18]
	0.58 (0.26–1.80) [b]	0.49 (0.24–1.87) [b]		18–89	Italy	[64]
	0.54 (0.5–0.57)	0.58 (0.54–0.61)		20–59	France	[8]
	1.23 (1.2–6.9) [c]			20–81	Japan	[65]
	0.43 (0.07–2.71)	0.358 (0.07–4.06)		30–75	USA	[44]
	0.27 (0.05–3.37)	0.48 (0.05–4.03)		31.1 ± 0.34	Finland	[33]
**Co**	0.37 (<0.2–1.1)	0.30 (<0.2–0.86)	0.33 (<0.2–1.1)	18–73	Australia	This study
			0.19 (0.04–0.8)	18–70	Germany	[18]
	0.35 (0.34–0.36)	0.30 (0.28–0.33)		20–59	France	[8]
**Cr**	<5	<1.7		18–73	Australia	This study
	0.64 (0.61–0.67)	0.56 (0.53–0.60)		20–59	France	[8]
	0.55 (0.5–41.8) [c]			20–81	Japan	[65]
**Cu**	930 (710–1420)	770 (650–950)	840 (650–1420)	18–73	Australia	This study
	1039 (986–1130)	894 (777–1046)		40 ± 2	Serbia	[52]
	862.2 (743.2–977.1) [a]	744.7 (661.9–822.6) [a]		12–60	China	[63]
	1070 (657–2004)	847 (532–1404)		18–49	Slovenia	[62]
			890 (712–1732)	18–60	Brazil	[57]
			1042 (720–1800)	18–70	Germany	[18]
**Hg**	1.8 <0.8–9.3)	2.1 (<0.8–7.7)	2.0 (<0.8–9.3)	18–73	Australia	This study
	3.31 (0.4–13.1)	5.92 (0.3–25.9)		12–78	Korea	[27]
	1.11 (<LOD–10.2)	1.25 (<LOD–31.0)		18–49	Slovenia	[62]
			1.4 (0.02–16)	18–70	Germany	[18]

	2.00 (1.90–2.09)	2.05 (1.94–2.16)		20–59	France	[8]
	2.20 (0.33–11.00)	2.85 (0.32–14.54)		31.1 ± 0.34	Finland	[33]
**I**	32.5 (20.1–82.7)	28.5 (19.5–44.6)	30.1 (19.5–82.7)	18–73	Australia	This study
	10.0 (5.7–17.6)	9.6 (4.5–19.5)	9.7 (4.54–19.5)	18–73	Australia	This study
	12.92 (9.98–16.35) [a]	10.05 (8.22–11.87) [a]		12–60	China	[63]
	12.29 (4.5–27.8)	10.90 (5.0–18.5)		12–78	Korea	[27]
	17.2 (5.83–40.5)	11.1 (5.69–11.4)		18–49	Slovenia	[62]
			9.6 (6.9–18.4)	18–60	Brazil	[57]
**Mn**			9.0 (4.8–18)	18–70	Germany	[18]
	8.56 (8.39–8.74)	7.59 (7.45–7.73)		20–59	France	[8]
	(5.2–19.8) [d]	(4.8–17.1) [d]	(4.9–18.9) [d]	20–64	USA	[66]
	13.2 (13.1–33.4) [c]			20–81	Japan	[65]
	9.57 (3.75–24.91)	8.95 (3.75–24.91)		30–75	USA	[44]
	12.9 (11.9–17.1)	13.4 (11.8–17.0)		40 ± 2	Serbia	[52]
**Mo**	0.51 (<0.3–1.7)	0.47 (<0.3–1.2)	0.49 (<0.3–1.7)	18–73	Australia	This study
			0.43 (0.06–4.0)	18–70	Germany	[18]
	2.0 (<2.0–4.0)	<2.0	2.0 (<2–4.0)	18–73	Australia	This study
	2.1 (<0.12–3.90)			18–60	Brazil	[57]
**Ni**			0.11 (<0.025–0.8)	18–70	Germany	[18]
	1.46 (1.42–1.51)	1.49 (1.43–1.54)		20–59	France	[8]
	1.81 (1.8–75.8) [c]			20–81	Japan	[65]
	13.2 (3.8–49.6)	13.9 (4.9–45.0)	13.6 (3.8–49.6)	18–73	Australia	This study
	38.4 (28.02–54.20) [a]	47.3 (34.75–62.34) [a]		12–60	China	[63]
	15.07 (1.2–36.2)	21.5 (1.8–58.1)		12–78	Korea	[27]
	16.7 (4.25–71.9)	19.3 (3.86–116)		18–49	Slovenia	[62]
			65.4 (5.1–163.0)	18–60	Brazil	[8]
**Pb**			22 (5–83)	18–70	Germany	[18]
	24.7 (12.3–68.9) [b]	44.4 (17.5–106) [b]		18–89	Italy	[64]
	18.9 (18.0–19.9)	26.9 (25.7–28.2)		20–59	France	[8]
	15.8 (15.7–105.0) [c]			20–81	Japan	[65]
	11.35 (1.4–74.7)	15.0 (4.2–59.6)		30–75	USA	[44]
	12.36 (0.80–91.9)	20.7 (3.9–145.5)		31.1 ± 0.34	Finland	[33]
	4.0 (2.8–5.9)	4.1 (2.6–6.2)	4.1 (2.6–6.2)	18–73	Australia	This study
**Sb**			<0.013 (<0.013–0.13)	18–70	Germany	[18]
	0.25 (0.03–0.46)	0.07 (0.05–0.08)		20–59	France	[8]
	140 (122–203)	142 (118–224)	141 (118–224)	18–73	Australia	This study
	94.6 (53.9–176)	115 (60.3–226)		18–49	Slovenia	[62]
**Se**			89.3 (68–245)	18–60	Brazil	[8]
			133 (85–182)	18–70	Germany	[18]
	(144.2–244.6) [d]	149.4–250.4) [d]	(152.3–251.3) [d]	20–64	USA	[66]
	97.9 (30–245)	109.1 (49.5–195.2)		31.1 ± 0.34	Finland	[33]

	81.8 (76.0–93.2)	84.8 (69.1–108)		40 ± 2	Serbia	[52]
**Tl**	0.061 (<0.06–0.10)	0.07 (<0.06–0.16)	0.06 (<0.06–0.16)	18–73	Australia	This study
			0.019 (<0.01–0.05)	18–70	Germany	[18]
	0.05 (0.04–0.05)	0.05 (0.04–0.05)		20–59	France	[8]
**U**	<0.1	<0.1		18–73	Australia	This study
			<0.003 (<0.003–0.006)	18–70	Germany	[18]
**V**	<0.45	<0.45		18–73	Australia	This study
**Zn**	6540 (4940–8390)	6900 (4620–9250)	6750 (4620–9250)	18–73	Australia	This study
	4764 (3730–6090) [a]	4564 (4392–4727) [a]		12–60	China	[63]
	6721 (3010–11733)	6494 (3400–10301)		18–49	Slovenia	[62]
	5554 (5503–5604)	6212 (6159–6264)		20–59	France	[8]
	4699 (4434–5185)	5706 (5385–6151)		40 ± 2	Serbia	[52]

[a] Geometric mean (percentile, P$_{25}$–P$_{75}$); [b] geometric mean (percentile, P$_{25}$–P$_{95}$); [c] geometric mean (median–max), [d] (percentile, P$_{2.5}$–P$_{97.5}$).

Supplementary Table S2. Concentration of trace elements in human blood plasma for both genders by country.

Element (μg/L)	Mean (range), female	Mean (range), male	Mean (range), female & male	All age group (y)	Country	Reference
**Al**	6.4 (<4–21.8)	7.4 (<4–81.8)	6.9 (4–82)	19–81	Australia	This study
			78.01 ± 14.48	40–70	Malaysia	[67]
			11.00 ± 8.99 (5.66–18.66) [a]	NP[b]	Switzerland	[26]
**Bi**	<0.08	<0.08		19–81	Australia	This study
**Co**	0.50 (0.22–1.34)	0.44 (0.21–1.14)	0.47 (0.21–1.3)	19–81	Australia	This study
			3.87 ± 1.63	40–70	Malaysia	[67]
			0.104 ± 0.158 (<0.1–0.155)	NP	Switzerland	[26]
**Cr**	<1.7	<1.7		19–81	Australia	This study
			6.43 ± 4.79	40–70	Malaysia	[67]
**Cu**	1240 (706–2490)	960 (672–1403)	1100 (670–2490)	19–81	Australia	This study
	757 (729–820)	644 (614–705)		40 ± 2	Serbia	[52]
	1089 ± 733 (711–1944)	857 ± 222 (637–1084)		NP	Switzerland	[26]
	1067 (530–2508)	926.4 (452.0–1425)		12–78	Korea	[27]
			1621 ± 711	40–70	Malaysia	[67]
**Mn**	1.1 (<1–3.1)	1.0 (<1–1.3)	1.0 (<1–3.1)	19–81	Australia	This study
	1.34 (1.34–2.05)	1.44 (1.35–2.08)		40 ± 2	Serbia	[52]
			0.40 ± 0.99 (<0.01–1.48)	NP	Switzerland	[26]
			14.23 ± 6.72	40–70	Malaysia	[67]
**Mo**	0.85 (0.27–2.67)	0.47 (<0.3–1.2)	0.91 (0.26–3.0)	19–81	Australia	This study
			0.44 ± 1.62 (<0.01–3.00)	NP	Switzerland	[26]
**Se**	124 (82–179)	130 (101–161)	130 (82–179)	19–81	Australia	This study
	63.8 (61.9–70.2)	66.3 (63.0–71.2)		40 ± 2	Serbia	[52]
			112.7 ± 32.3 (87.3–143.5)	NP	Switzerland	[26]
	110 (70.4–172)	117 (55–207)		12–78	Korea	[27]

**Tl**	0.11 (0.05–0.20)	0.10 (0.04–0.20)	0.11 (0.04–0.20)	19–81	Australia	This study
			<0.2 (<0.2–0.21)	15	Sweden	[24]
**V**	0.20 (<0.2–0.22)	0.20 (<0.2–0.22)	0.2 (0.2–0.22)	19–81	Australia	This study
**Zn**	1140 (850–1660)	1160 (819–1496)	1150 (820–1660)	19–81	Australia	This study
	535 (508–583)	591 (574–648)		40 ± 2	Serbia	[52]
	884 (542–2415)	902 (450–1689)		12–78	Korea	[27]
			1478 ± 583	40–70	Malaysia	[67]
			810 ± 223 (637–1004)	NP	Switzerland	[26]

[a] Percentile range (P_{25}–P_{95}), [b] NP—not provided for age range.
